# Amanita Mushroom Toxin Poisoning in Los Angeles County

**DOI:** 10.14309/crj.0000000000001246

**Published:** 2023-12-28

**Authors:** Parker G. Jobin, Connor Stewart, Aarshi Vipani, Ingrid Perez-Alvarez, Samuel Pepkowitz, Ellen Klapper, Anders Berg, Kaytlena Stillman, Sam Torbati, Alexander Kuo, Hirsh Trivedi, Ju Dong Yang, Jonathan Steinberger, Richard J. Van Allan, Oren Friedman, Kathryn Cardoza, Walid S. Ayoub

**Affiliations:** 1 Department of Medicine, Cedars-Sinai Medical Center, Los Angeles, CA; 2 Karsh Division Gastroenterology & Hepatology, Cedars-Sinai Medical Center, Los Angeles, CA; 3 Department of Pathology and Laboratory Medicine, Cedars-Sinai Medical Center, Los Angeles, CA; 4 Department of Emergency Medicine, Cedars-Sinai Medical Center, Los Angeles, CA; 5 Comprehensive Transplant Center, Cedars-Sinai Medical Center, Los Angeles, CA; 6 Department of Imaging, Cedars-Sinai Medical Center, Los Angeles, CA; 7 Division of Pulmonary & Critical Care Medicine, Cedars-Sinai Medical Center, Los Angeles, CA

**Keywords:** Amatoxin, mushroom poisoning, silibinin, acute liver failure, case report

## Abstract

Mushroom (amatoxin) poisoning from ingestion is a rare but life-threatening medical emergency characterized by gastrointestinal symptoms before progression to multisystem organ failure in severe cases. Many therapies of amatoxin intoxication have been described, including supportive care, medical therapies, detoxification strategies, and liver transplant. The evidence supporting these therapies remains limited due to the rarity of amatoxin poisoning and challenge of a timely diagnosis. We report a case of amatoxin poisoning in Los Angeles causing severe liver injury without acute liver failure treated successfully using medical therapies, gallbladder drainage, and plasma exchange.

## INTRODUCTION

Mushroom poisoning is a potentially life-threatening emergency associated with significant morbidity and mortality.^1^ The *Amanita phalloides* mushroom is responsible for most fatalities caused by mushroom poisoning.^2^ The cyclopeptides amatoxin and phallotoxin contribute to mushroom-related toxicity. While phallotoxin impairs cellular membranes in the gastrointestinal lining resulting in the initial presentation of gastroenteritis-like symptoms including diarrhea, abdominal cramping, nausea, and vomiting, amatoxin is responsible for acute hepatic and renal failure through direct toxicity to hepatocytes and tubule cells due to inhibition of RNA polymerase II.^1^ There were 95 cases of cyclopeptide-containing mushroom exposures in the United States during the year 2021.^3^ We report a case of amatoxin poisoning leading to severe acute liver injury in Los Angeles County.

## CASE REPORT

**Figure 1.**
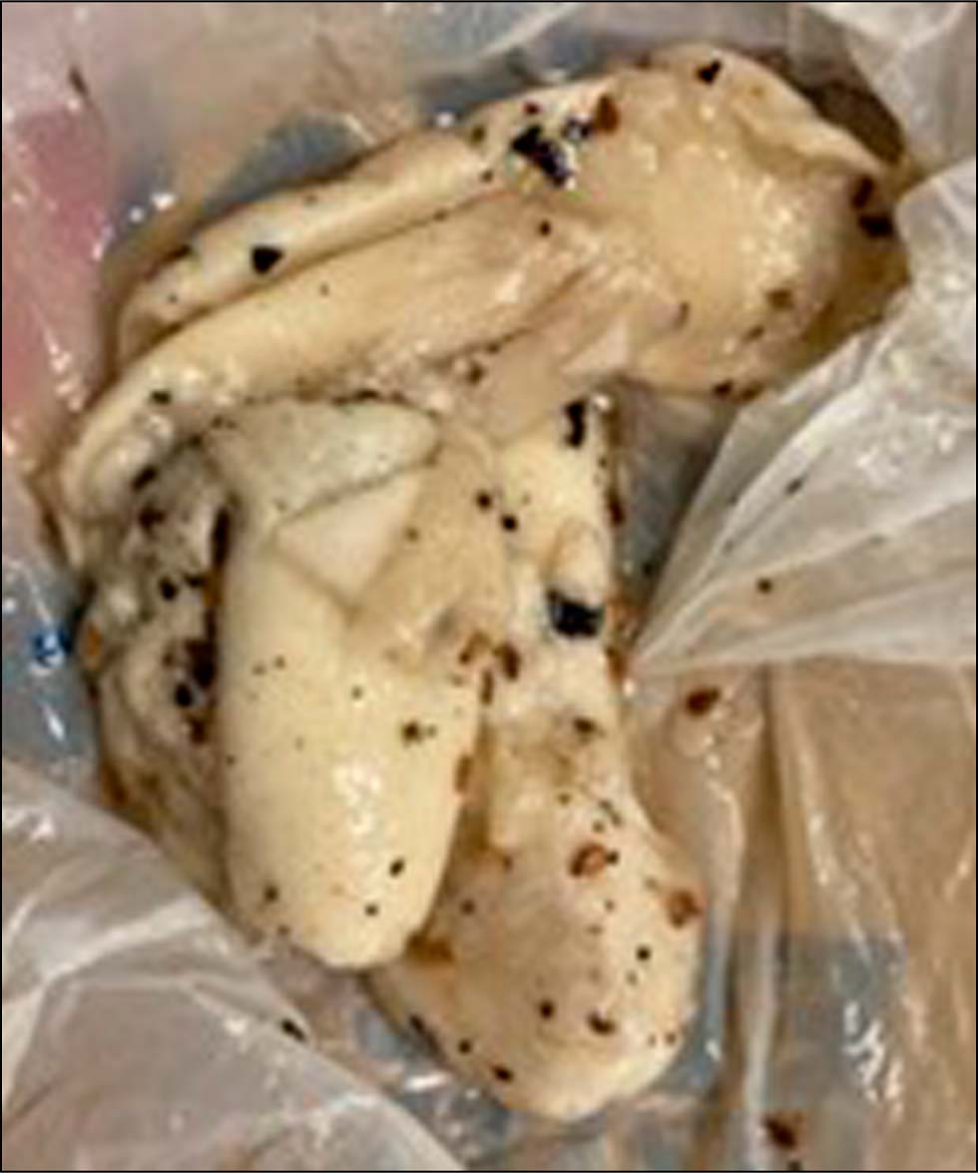
Picture of mushrooms foraged by the patient. The patient's family brought the remaining mushrooms the patient foraged and fried which she did not consume.

**Figure 2.**
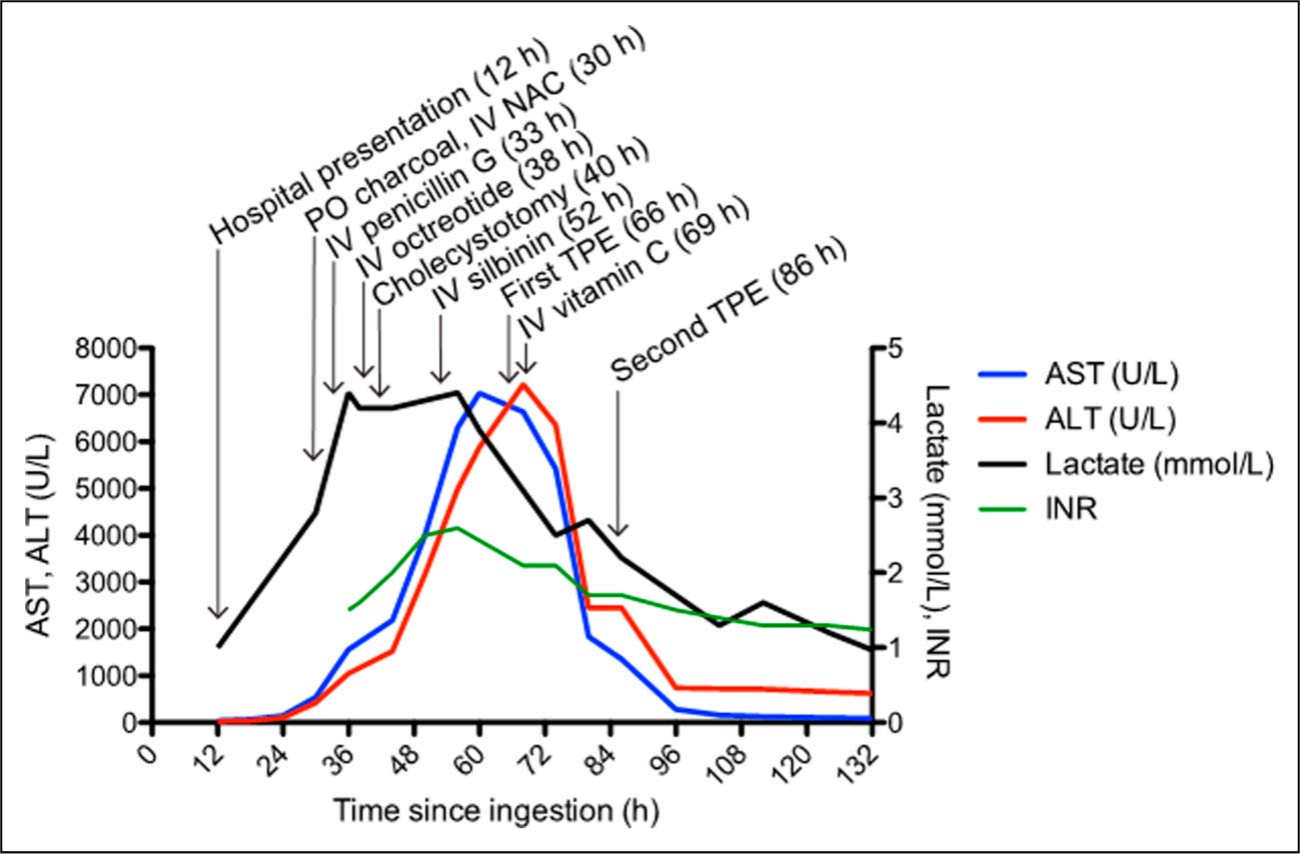
Patient's clinical course since the time of ingestion. Progression of laboratory tests in relation to clinical interventions since the estimated time of mushroom ingestion to 132 hours (h), after which time all test values continued to decrease until the day of discharge on day 11. ALT, alanine aminotransferase; AST, aspartate aminotransferase; INR, international normalized ratio; IV, intravenous; NAC, N-acetylcysteine; PO, per oral; TPE, therapeutic plasma exchange.

A 76-year-old woman with a medical history of hypertension and type 2 diabetes mellitus presented with 6 hours of abdominal cramping, nausea, vomiting, and diarrhea. Twelve hours before symptoms onset, she consumed 5 white mushrooms that she foraged from Temescal Canyon (Figure 1). Initial testing revealed unremarkable liver aminotransferases: alkaline phosphatase 121 U/L, aspartate aminotransferase (AST) 45 U/L, and alanine aminotransferase (ALT) 33 U/L. However, her aminotransferases increased to AST and ALT of 148 U/L and 101 U/L, respectively, within 12 hours (Figure 2). She had a negative workup for acetaminophen toxicity, acute viral hepatitis, autoimmune hepatitis, parasitic gastrointestinal infection, and clostridium difficile infection (Table 1). She had a low ceruloplasmin with elevated 24-hour urine copper attributed to acute liver injury. Contrast computed-tomography of the abdomen revealed no signs of pancreatitis or Budd-Chiari syndrome. Therefore, given the temporal association of mushroom ingestion with her symptoms, her presentation was deemed likely secondary to amatoxin poisoning. She was admitted while displaying normal vitals and mental status.

**Table 1.** Selected laboratory results

Study	Result
Acetaminophen, serum	<18 mg/dL, negative
Hepatitis A IgM	Negative
Hepatitis B sAg	Negative
Hepatitis B sAb IgG	Positive
Hepatitis B cAb IgG	Positive
Hepatitis C Ab	Negative
CMV PCR	Undetectable
EBV PCR	Undetectable
VZV PCR	Undetectable
ANA	<40 titer, negative
Antismooth muscle Ab	<20 titer, negative
lgG, serum	927 mg/dL, normal
Ceruloplasmin	18 mg/dL, decreased
24-hour urine copper	289 μg/24 hr, elevated
Lipase	57 U/L, negative
GI stool pathogen PCR	No detected organism
*C. difficile* stool PCR	Negative

Ab, antibody; ANA, antinuclear antibody; cAb, core antibody; CMV, cytomegalovirus; EBV, Epstein-Barr virus; IgG, immunoglobulin G; IgM, immunoglobulin M; GI, gastrointestinal; PCR, polymerase chain reaction; sAb, surface antibody; sAg, surface antigen; VZV, varicella zoster.

Poison control was consulted regarding treatment decisions. The patient received intravenous (IV) fluid boluses. Activated charcoal was administered for absorption elimination enhancement but subsequently held for vomiting. IV N-acetylcysteine (NAC), an antioxidant with evidence for improved outcomes in amatoxin poisoning, was administered.^1^ The first-line therapy for amatoxin toxicity, silibinin, was emergently dispatched to our facility.^4,5^ Silibinin is an amatoxin hepatocyte uptake inhibitor that lowers mortality when given within 24 hours of ingestion. In the interim, IV penicillin G was administered as an alternative amatoxin uptake inhibitor.^2^

Her aminotransferases rose to AST of 1,565 of U/L and ALT of 1,053 U/L the morning of her second day of hospitalization (42 hours after ingestion). Lactate rose to 4.2 mmol/L and international normalized ratio increased as well to 1.5. A hepatology consultation was requested, and temporizing measures of amatoxin detoxification were implemented, given the patient's worsening biochemical status. IV octreotide was administered to minimize gallbladder contraction^6,7^ and slow enterohepatic recirculation of amatoxin. Subsequently, a percutaneous cholecystostomy drain was placed to remove amatoxin-laden bile. The bile obtained from the cholecystostomy tested positive for amatoxin (data not shown).^8^ IV silibinin was initiated on arrival on day 3 of hospitalization (58 hours after ingestion), and penicillin G was discontinued.

The patient's aminotransferases peaked shortly after silibinin administration at AST and ALT of 7,037 and 5,883 U/L, respectively. Hepatology recommended therapeutic plasma exchange (TPE) as a further temporizing measure for amatoxin detoxification. She underwent 2 TPE procedures with 5% albumin and fresh frozen plasma as replacement fluid on 72 and 92 hours after ingestion. After TPE, her aminotransferases rapidly decreased. We began IV ascorbic acid as an additional antioxidant. The patient remained hemodynamically stable and did not develop hepatic encephalopathy. Her lactate and international normalized ratio returned to normal range 3 and 4 days after ingestion, respectively. The patient's remaining hospital course was marked by liver enzyme improvement. Her AST normalized by day 9 after ingestion, but her ALT remained elevated to 155 U/L by discharge day (day 11).

## DISCUSSION

Amatoxin poisoning is a rare but serious emergency in which presentation varies from mild intoxication to lethality.^1,9^ Patients present 6–12 hours after ingestion with symptoms including diarrhea, abdominal cramping, nausea, and vomiting followed by a respite of masked recovery of 24–36 hours.^9,10^ During this period, amatoxin travels through enterohepatic and systemic circulation damaging both the liver and kidney.^11^ Severe cases may progress to acute liver failure within 48–96 hours after ingestion often coupled with acute renal failure.^1^

Recognizing and treating amatoxin poisoning is challenging given its rarity. Strategies include medical therapies, detoxification, and liver transplant in fulminant cases.^1,9,12^ Supportive care including adequate fluid resuscitation, oral activated charcoal for elimination enhancement, hepatic support with lactulose, and management of comorbidities develop including renal failure and sepsis. These approaches have decreased historical mortality rates of 50% to less than 10%.^13^

Therapeutic agents for amatoxin toxicity include amatoxin uptake inhibitors and antioxidants. Of the amatoxin uptake inhibitors, high-dose penicillin G was initiated initially while awaiting receipt of silibinin. Antioxidants including NAC, ascorbic acid, and cimetidine are also recommended for limiting known hepatotoxic oxidizing properties of amatoxin, but only NAC has evidence for improved outcomes.^4^ The treatment in the most severe cases of acute liver failure is liver transplant.^1,2,12^

Temporizing measures for detoxification have been attempted in cases of moderate to severe hepatic injury with limited evidence. Systemic removal methods including TPE have been reported to improve hepatic function in small trials.^12,14,15^ TPE has been reported as an effective therapy for amatoxin poisoning, albeit based on case series, which compared outcomes to that of historical controls, and observational studies.^12,14^ Early initiation of TPE within 48 hours of ingestion is recommended.^16^ Our rationale for TPE in this patient was driven by rising aminotransferases despite initiating silibinin. Nasobiliary catheter drainage may be helpful within 24 hours of ingestion when other treatments are unavailable.^2^ Gallbladder drainage to remove amatoxin-laden bile has also been reported in cases.^17,18^ While octreotide has been proposed to slow enterohepatic recirculation by inhibiting gallbladder contraction, there is minimal evidence in human and nonhuman cases.^6,7^ Our use of gallbladder drainage with octreotide was influenced by worsening biochemical values while awaiting silibinin. Ultimately, our bridging strategy to avoid the sequelae of acute liver failure depended on expert advice, which should be obtained from clinical hepatologists and toxicologists.

## DISCLOSURES

Author contributions: PG Jobin drafted the initial manuscript. C. Stewart, A. Vipani, I. Perez-Alvarez, S. Pepkowitz, E. Klapper, A. Berg, K. Stillman, S. Torbati, A. Kuo, H. Trivedi, JD Yang, J. Steinberger, RJ Van Allan, O. Friedman, K. Cardoza, and WS Ayoub critically revised the manuscript for intellectual content. WS Ayoub is the article guarantor.

Financial disclosure: None to report.

Informed consent was obtained for this case report.

## References

[1] Broussard CN Aggarwal A Lacey SR . Mushroom poisoning—From diarrhea to liver transplantation. Am J Gastroenterol. 2001;96(11):3195–8. 11721773 10.1111/j.1572-0241.2001.05283.x

[2] Karvellas CJ Tillman H Leung AA . Acute liver injury and acute liver failure from mushroom poisoning in North America. Liver Int. 2016;36(7):1043–50. 26837055 10.1111/liv.13080

[3] Gummin DD Mowry JB Beuhler MC . 2021 annual report of the National Poison Data System (NPDS) from America's Poison Centers: 39th annual report. Clin Toxicol. 2022;60(12):1381–643. 10.1080/15563650.2022.2132768 36602072

[4] Poucheret P Fons F Doré JC Michelot D Rapior S. Amatoxin poisoning treatment decision-making: Pharmaco-therapeutic clinical strategy assessment using multidimensional multivariate statistic analysis. Toxicon. 2010;55(7):1338–45. 20152849 10.1016/j.toxicon.2010.02.005

[5] Ganzert M Felgenhauer N Schuster T Eyer F Gourdin C Zilker T. Knollenblätterpilzvergiftung. Dtsch Med Wochenschr. 2008;133(44):2261–7. 18946850 10.1055/s-0028-1091268

[6] Mackenzie CA Austin E Thompson M Tirona RG. Cyclosporine as a novel treatment for amatoxin-containing mushroom poisoning: A case series. Toxicol Commun. 2022;6(1):23–7.

[7] Goupil RC Davis M Kaufman A Roberts D Mitchell T. Clinical recovery of 5 dogs from amatoxin mushroom poisoning using an adapted Santa Cruz protocol for people. J Vet Emerg Crit Care. 2021;31(3):414–27. 10.1111/vec.13040 33458945

[8] Bever CS Swanson KD Hamelin EI . Rapid, sensitive, and accurate point-of-care detection of lethal amatoxins in urine. Toxins. 2020;12(2):123. 32075251 10.3390/toxins12020123 PMC7076753

[9] Diaz JH. Nephrotoxic mushroom poisoning: Global epidemiology, clinical manifestations, and management. Wilderness Environ Med. 2021;32(4):537–44. 34629291 10.1016/j.wem.2021.09.002

[10] Diaz JH. Syndromic diagnosis and management of confirmed mushroom poisonings. Crit Care Med. 2005;33(2):427. 15699849 10.1097/01.ccm.0000153531.69448.49

[11] Larrey D Pageaux GP. Hepatotoxicity of herbal remedies and mushrooms. Semin Liver Dis. 1995;15(3):183–8. 7491502 10.1055/s-2007-1007276

[12] Kieslichova E Frankova S Protus M . Acute liver failure due to Amanita phalloides poisoning: Therapeutic approach and outcome. Transpl Proc. 2018;50(1):192–7. 10.1016/j.transproceed.2017.11.032 29407307

[13] De Olano J Wang JJ Villeneuve E . Current fatality rate of suspected cyclopeptide mushroom poisoning in the United States. Clin Toxicol. 2021;59(1):24–7. 10.1080/15563650.2020.1747624 32237919

[14] Jander S Bischoff J. Treatment of Amanita phalloides poisoning: I. Retrospective evaluation of plasmapheresis in 21 patients. Ther Apher. 2000;4(4):303–7. 10975478 10.1046/j.1526-0968.2000.004004303.x

[15] Zhang J Zhang Y Peng Z . Experience of treatments of Amanita phalloides-induced fulminant liver failure with molecular adsorbent recirculating system and therapeutic plasma exchange. ASAIO J. 2014;60(4):407–12. 24727538 10.1097/MAT.0000000000000083

[16] Padmanabhan A Connelly-Smith L Aqui N . Guidelines on the use of therapeutic apheresis in clinical practice: Evidence-based approach from the Writing Committee of the American Society for Apheresis: The Eighth Special Issue. J Clin Apher. 2019;34(3):171–354. 31180581 10.1002/jca.21705

[17] Dutta A Das AK Pegu AK Bipul CK Mitchell T. Ultrasoundguided percutaneous transhepatic gall bladder aspiration (PTGBA) in the treatment of mushroom poisoning. J Indian Soc Toxicol. 2018;14(1):1–5.

[18] Mitchell S Deori P Long C. Ultrasound guided simple gallbladder aspiration for amatoxin mushroom poisoning (AMP) induced hepatoxicity and fulminant hepatic failure: An effective treatment alternative when IV silibinin is unavailable or likely to fail. In: APAMT 2016.

